# Meckel’s Diverticulum Causing Small-Bowel Volvulus in an Elderly Patient: A Case Report

**DOI:** 10.7759/cureus.107792

**Published:** 2026-04-27

**Authors:** Nona Shalisha Sallay, Sanura Malinda Pallegoda Vithana, H.L.D.S. Ariyaratne

**Affiliations:** 1 General Surgery, Colombo South Teaching Hospital, Nugegoda, LKA; 2 Surgery, National Hospital of Sri Lanka, Colombo, LKA

**Keywords:** acute abdomen, elderly patient, intestinal obstruction, meckel’s diverticulum, small bowel volvulus

## Abstract

Meckel’s diverticulum (MD) represents a remnant of the embryonic vitelline duct and is recognized as a congenital anomaly of the small intestine. While it is more frequently identified in children, the majority of cases remain clinically silent, with symptomatic presentation in adults being relatively rare. When present, symptoms typically reflect complications, including gastrointestinal hemorrhage, diverticulitis, or intestinal obstruction. The probability of developing complications decreases with advancing age, making clinically significant presentations in elderly individuals uncommon and diagnostically challenging.

We present the case of a 72-year-old male with features of small bowel obstruction, in whom intraoperative findings demonstrated a small-bowel volvulus occurring around an MD that functioned as the axis of rotation.

## Introduction

Meckel’s diverticulum (MD) is a congenital remnant arising from the persistence of the vitelline (omphalomesenteric) duct during fetal development [1-3]. It represents a true diverticulum, comprising all layers of the intestinal wall, and is traditionally characterized by the so-called *rule of 2s* [2,4]. Although it is estimated to occur in approximately 2% of the population, the majority of individuals remain asymptomatic. Clinical manifestations are more commonly observed in childhood, while presentation in adults is uncommon and often incidental [1,3,5].

Complications arise in a minority of patients, encompassing gastrointestinal bleeding, inflammation, and intestinal obstruction [3,6]. In adults, intestinal obstruction is the most prevalent manifestation and may result from mechanisms such as intussusception, volvulus, or fibrous bands associated with the diverticulum [6-8]. Small-bowel volvulus, characterized by the twisting of the bowel around its mesentery, is an infrequent yet significant cause of intestinal obstruction, especially when resulting from mesenteric disease [8].

Preoperative diagnosis remains difficult because clinical and radiological findings are not sufficiently specific, and a definitive diagnosis is often made during the operation [1,7,9]. Surgical resection is the primary treatment for symptomatic cases, whereas the management of incidentally identified MD is still debated [10-12].

Small-bowel volvulus with the diverticulum acting as the axis of rotation is an exceptionally rare mechanism, particularly in adult patients, and poses significant diagnostic challenges. This case highlights this uncommon presentation and underscores the importance of early surgical intervention.

## Case presentation

A 72-year-old male was admitted with a 48-hour history of colicky central abdominal pain, worsening abdominal distension, bilious vomiting, and constipation. He had a background of well-controlled hypertension and type 2 diabetes mellitus and no previous abdominal surgical interventions.

Physical examination

On examination, the patient appeared mildly dehydrated but was hemodynamically stable. The abdomen was markedly distended and tympanic, with diffuse tenderness but no signs of peritonism. Bowel sounds were exaggerated. Digital rectal examination revealed an empty rectum, and no external hernias were detected.

Diagnostic workup

Laboratory investigations demonstrated mild leukocytosis (12.5 × 10⁹/L) and hypokalemia (3.2 mmol/L), consistent with vomiting and third-space losses.

**Figure 1:**
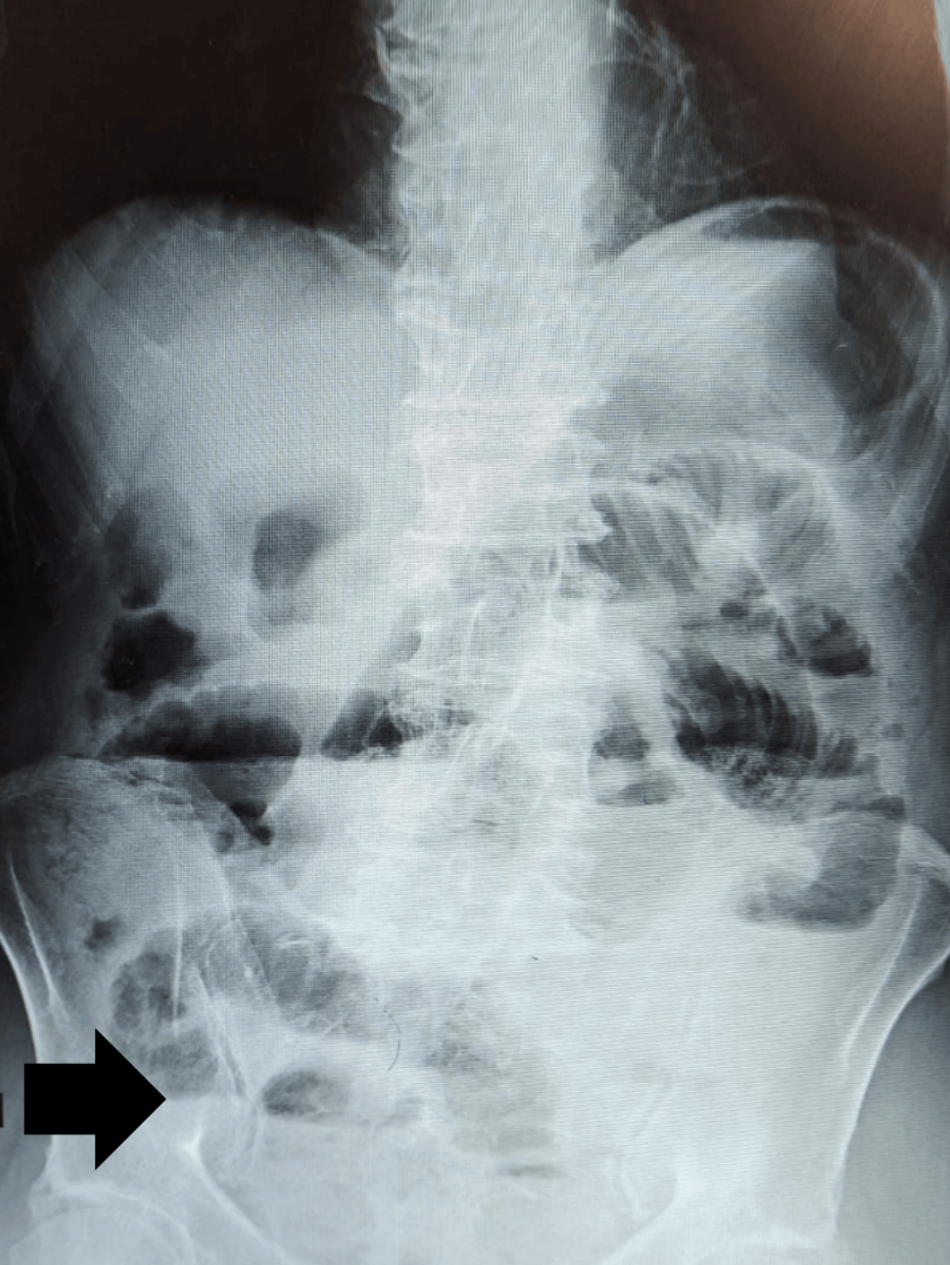
Erect abdominal radiograph demonstrating multiple air–fluid levels. The black arrow highlights a representative air–fluid interface.

**Figure 2:**
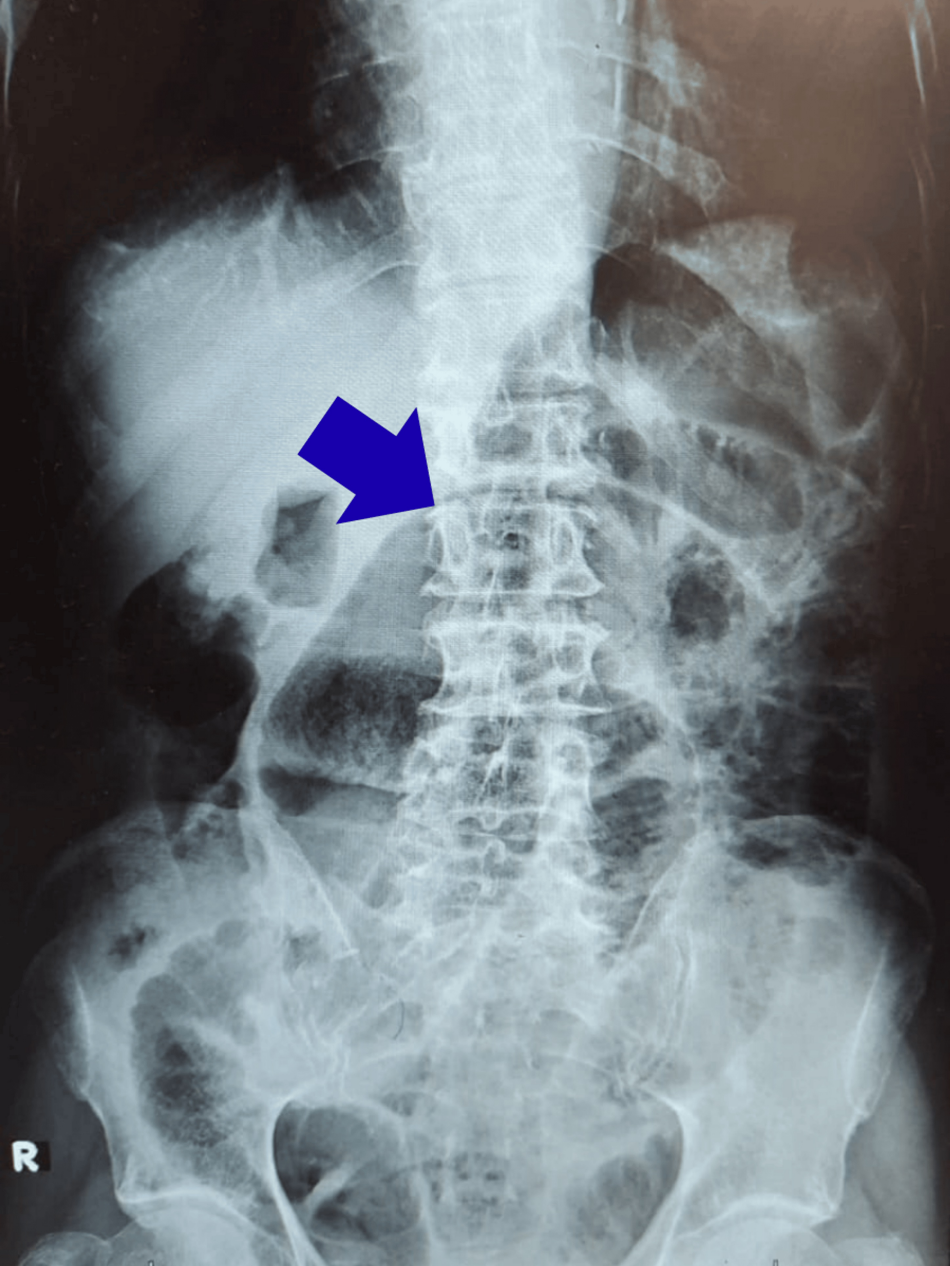
Supine abdominal X-ray showing dilated bowel loops. The blue arrow indicates a dilated bowel loop.

The erect abdominal X-ray (Figure 1) demonstrated multiple air-fluid levels, whereas the supine abdominal X-ray (Figure 2) revealed dilated bowel loops.

**Figure 3:**
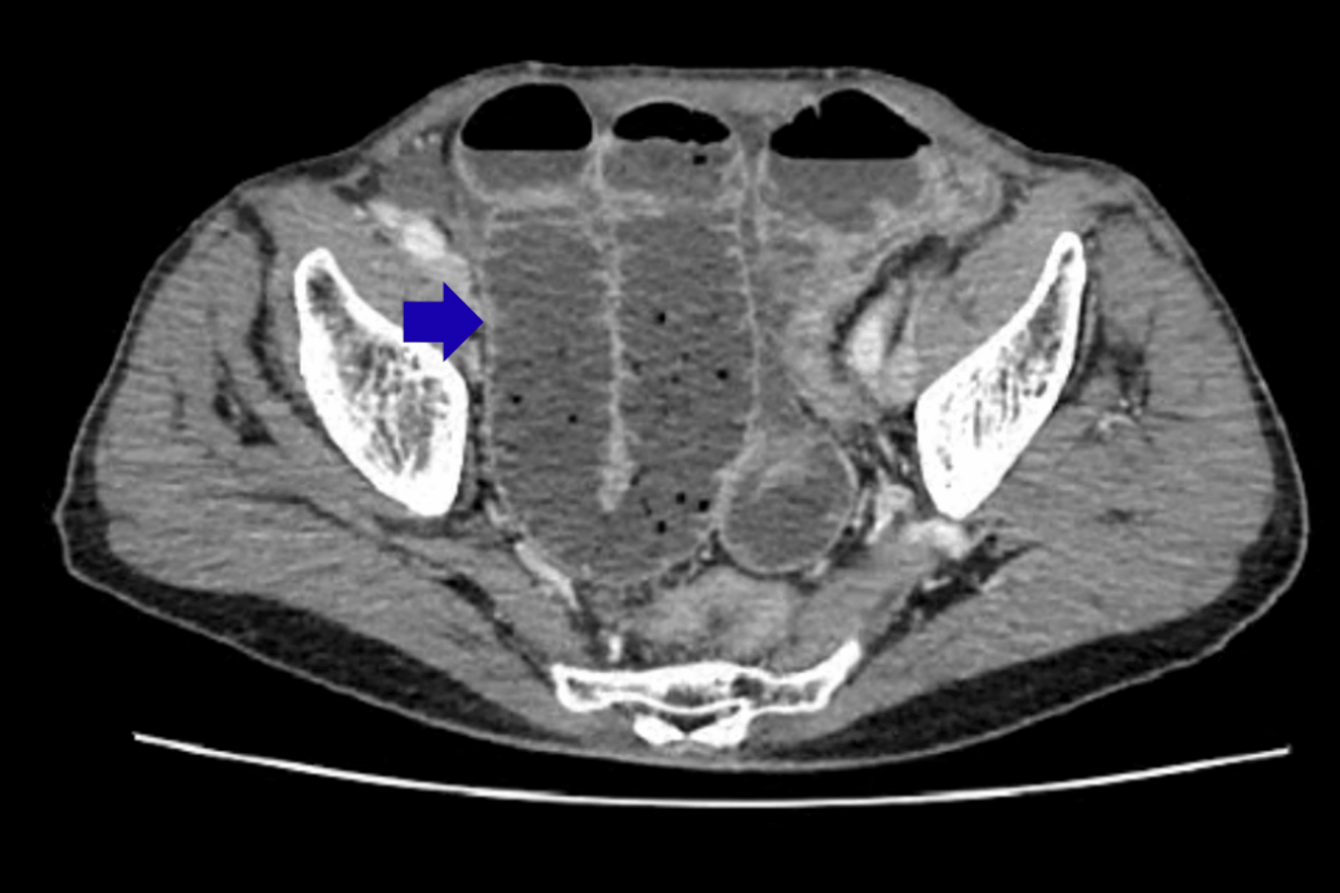
Contrast-enhanced CT (CECT) of the abdomen, axial section, demonstrating small-bowel volvulus (blue arrow).

Contrast-enhanced CT of the abdomen and pelvis revealed features consistent with small bowel obstruction, with a distinct transition point identified in the distal ileum and associated dilatation of the proximal bowel loops (Figure 3).

However, the underlying cause - specifically an MD or internal band - was not identified preoperatively.

Therapeutic intervention

Given the high-grade obstruction and evolving clinical picture, the patient underwent emergency exploratory laparotomy.

**Figure 4:**
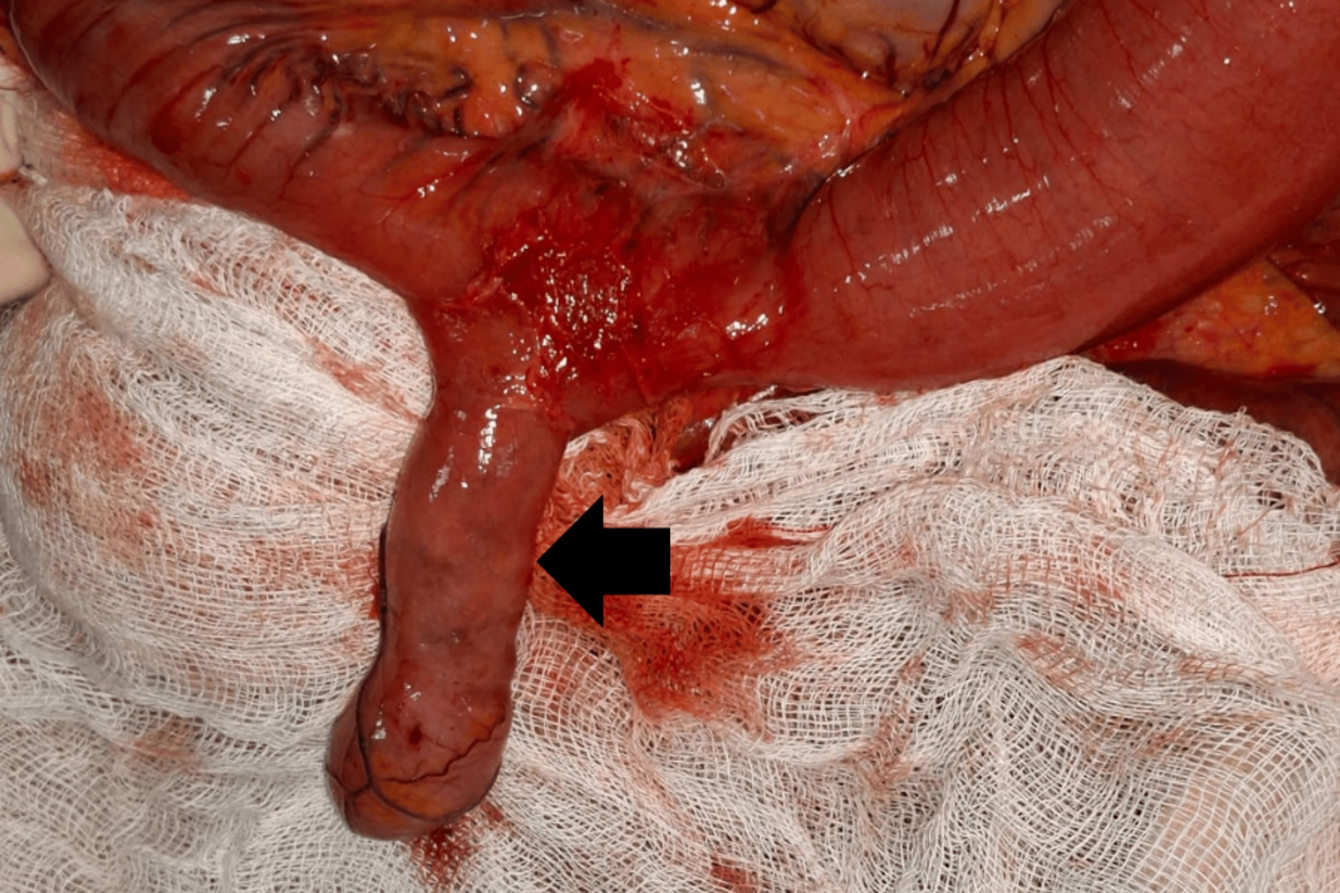
Intraoperative image showing Meckel’s diverticulum arising from the antimesenteric border of the ileum, with adjacent dilated bowel loops. The black arrow indicates Meckel’s diverticulum.

Intraoperatively, markedly dilated proximal small bowel loops were noted. A 4 cm MD was identified on the antimesenteric border approximately 60 cm proximal to the ileocecal valve (Figure 4).

A persistent fibrous (vitelline) band extended from the tip of the diverticulum to the adjacent mesentery, forming a mesenteric defect. Distal ileal loops had herniated through this defect and twisted around the diverticulum, resulting in small-bowel volvulus with the diverticulum acting as the axis of rotation.

There was no evidence of bowel ischemia, gangrene, or perforation.

**Figure 5:**
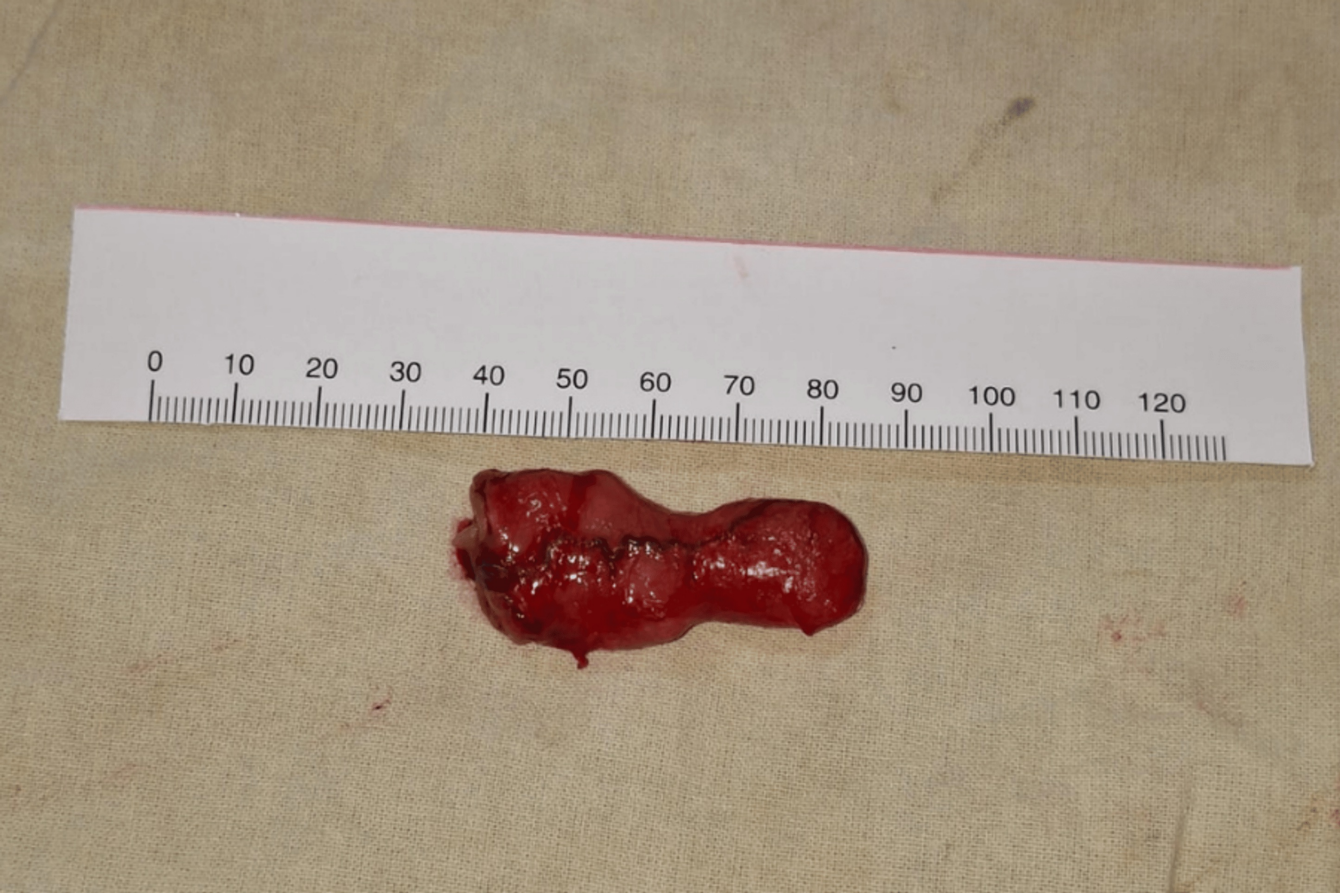
Resected Meckel’s diverticulum measuring approximately 4 cm.

The fibrous band was divided, relieving the volvulus. Given the narrow base of the diverticulum and the absence of surrounding ileal involvement, a wedge diverticulectomy (Figure 5) was performed using a linear stapler. The staple line was reinforced with interrupted seromuscular sutures.

Outcome and follow-up

The postoperative course was uneventful. Bowel function returned, and oral intake resumed on postoperative day 4. The patient was discharged on postoperative day 7 in good condition.

At six-week follow-up, he remained asymptomatic with normal bowel habits.

Histopathology

**Figure 6:**
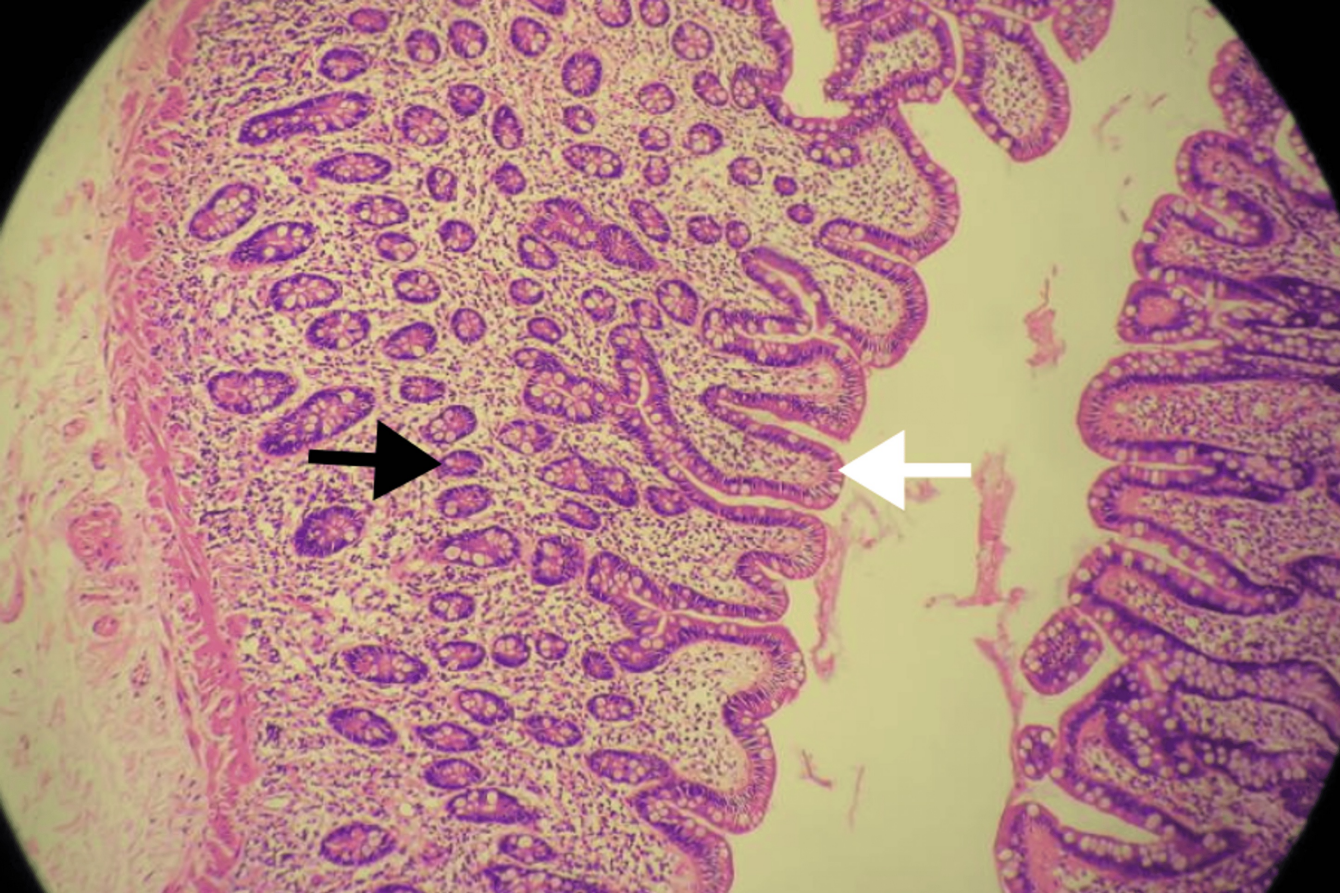
Histology slide of Meckel’s diverticulum demonstrating intestinal-type mucosa with villi (white arrow) and intestinal glands (black arrow) (hematoxylin and eosin stain, ×100 magnification).

Histopathological examination confirmed a true diverticulum containing all layers of the intestinal wall. No ectopic gastric or pancreatic mucosa was identified, and there was no evidence of malignancy (Figure 6).

## Discussion

Epidemiology and clinical significance

Despite an estimated prevalence of about 2%, MD remains asymptomatic in most individuals, with complications arising in only a small subset, corresponding to a lifetime risk of approximately 4% [1,3]. The likelihood of complications decreases with advancing age, making symptomatic presentation in adults uncommon and diagnostically challenging [1,5]. Despite its embryological prevalence, MD remains a diagnostic challenge in adult surgical practice.

Clinical presentation and pathophysiology

The clinical presentation of MD varies with age. While gastrointestinal bleeding is more frequently observed in children, adult patients more commonly present with intestinal obstruction or diverticulitis [3,6,9]. Among adults, intestinal obstruction is the most common mode of presentation. It may result from a variety of mechanisms such as intussusception, internal hernias, volvulus, or compression related to fibrous bands [6-8]. Adhesive or mesodiverticular bands may trap adjacent bowel loops, resulting in mechanical obstruction [7].

Mechanism in the present case

In the present case, small bowel obstruction resulted from volvulus, with the MD acting as the axis for rotation of the bowel. This mechanism is particularly rare and has been infrequently described in the literature [8]. The diverticulum may function as a fixed pivot point, allowing adjacent bowel loops to twist around it, potentially leading to closed-loop obstruction and bowel ischemia if not promptly recognized and treated.

Diagnostic challenges

In adults, MD is frequently difficult to diagnose because its presentation is nonspecific and can mimic common surgical pathologies such as appendicitis or small bowel obstruction [7,9]. Radiological investigations, particularly computed tomography, may demonstrate indirect features such as a transition point or whirl sign; however, identification of the diverticulum as the underlying cause is frequently inconclusive [8-9]. Accordingly, confirmation of the diagnosis is often achieved intraoperatively, underscoring the importance of maintaining clinical awareness in cases of small bowel obstruction, especially in the absence of previous abdominal surgical interventions.

Management and surgical approach

Surgical resection remains the cornerstone of management for symptomatic MD. The choice of procedure depends on intraoperative findings, including inflammation, ischemia, and involvement of the diverticular base. In this case, segmental bowel resection was performed due to volvulus and the associated risk of compromised bowel viability, consistent with current recommendations favoring resection in complicated MD [11]. This approach ensures complete removal of diseased tissue and minimizes the risk of recurrence.

Management of incidentally discovered MD

The management of incidentally discovered MD remains controversial. Evidence suggests that prophylactic resection should be considered in selected patients with risk factors such as younger age, male sex, diverticulum length greater than 2 cm, and the presence of abnormal or ectopic tissue [10]. In the absence of these risk factors, routine resection is not universally recommended, and management should be individualized [11-12].

The present case illustrates an infrequent but relevant etiology of small bowel obstruction in adults. Consideration of MD is warranted when evaluating intestinal obstruction, especially in individuals with no previous abdominal surgical history. Early surgical intervention is essential to prevent complications such as bowel ischemia and perforation. Increased awareness of this uncommon mechanism may facilitate timely diagnosis and enhance patient outcomes.

## Conclusions

Although uncommon in adults, MD should be recognized as a clinically relevant cause of small bowel obstruction. Volvulus with the diverticulum acting as the axis of rotation represents a rare and diagnostically challenging mechanism. The present case emphasizes the relevance of considering MD when evaluating patients with features of intestinal obstruction, particularly in the absence of prior abdominal surgery. Prompt surgical intervention remains essential to prevent serious complications, including bowel ischemia and perforation. Greater awareness of this atypical presentation may improve diagnostic accuracy and patient prognosis.
